# Rare primary dyslipidaemias associated with low LDL and HDL cholesterol values in Portugal

**DOI:** 10.3389/fgene.2022.1088040

**Published:** 2023-04-17

**Authors:** Ana Catarina Alves, Beatriz Miranda, Oana Moldovan, Raquel Espírito Santo, Raquel Gouveia Silva, Sandra Soares Cardoso, Luísa Diogo, Mónica Seidi, Silvia Sequeira, Mafalda Bourbon

**Affiliations:** ^1^ Grupo de Investigação Cardiovascular, Unidade de Investigação e Desenvolvimento, Departamento de Promoção da saúde e doenças não transmissíveis, Instituto Nacional de Saúde Doutor Ricardo Jorge, Lisboa, Portugal; ^2^ BioISI—Biosystems & Integrative Sciences Institute, Faculty of Sciences, University of Lisboa, Lisboa, Portugal; ^3^ Serviço de Genética Médica, Departamento de Pediatria, Hospital de Santa Maria, CHULN E P E, Centro Académico de Medicina de Lisboa, Lisboa, Portugal; ^4^ Serviço de Endocrinologia, Hospital de Loulé, Loulé, Portugal; ^5^ Serviço de Pediatria, Centro Hospitalar Tondela-Viseu, Viseu, Portugal; ^6^ Centro de Referência de Doenças Hereditárias Do Metabolismo, Hospital Pediátrico—Centro Hospitalar Universitário de Coimbra, Coimbra, Portugal; ^7^ Serviço de Medicina Interna, Hospital de Santo Espírito de Angra Do Heroísmo, Angra Do Heroísmo, Portugal; ^8^ Centro de Referência de Doenças Hereditárias Do Metabolismo, Hospital de Dona Estefânia—Centro Hospitalar Universitário de Lisboa Central, Lisboa, Portugal

**Keywords:** rare dyslipidaemias, LDL cholesterol, HDL cholesterol, hypobetalipoproteinemia, hypoalphalipoproteinemia

## Abstract

**Background:** Dyslipidaemia represents a group of disorders of lipid metabolism, characterized by either an increase or decrease in lipid particles, usually associated with triglycerides, LDL cholesterol (LDL-C) and/or HDL cholesterol (HDL-C). Most hyperlipidaemias and HDL deficiencies confer an increased cardiovascular risk, while hypolipidaemia, such as abeta or hypobetalipoproteinemia, may present different manifestations ranging from poor weight progression to neurological manifestations. The aim of this study is to present 7 cases with rare dyslipidaemias associated with low LDL or low HDL cholesterol values, referred to our laboratory for the genetic identification of the cause of the dyslipidaemia.

**Methods:** Lipid profile was determined for each individual in an automated equipment Integra Cobas (Roche). Molecular analysis was performed by NGS with a target panel of 57 genes involved in lipid metabolism (Sure select QXT, Agilent) and samples were run in a NextSEQ Sequencer (Illumina). Only genes associated to rare forms of low HDL-c or LDL-c were analysed for this work, namely: *ABCA1*, *APOA1*, *LCAT, SCARB1, APOB, PCSK9, MTTP, SAR1B, and ANGPTL3*. All rare variants (MAF<5%) found in these genes were confirmed by Sanger sequencing.

**Results and discussion:** This study includes 7 index cases (IC), with the following clinical diagnoses: Fish Eye Disease (1), Hypoalphalipoproteinemia (1) and Abetalipoproteinemia (ABL) / Familial Hypobetalipoproteinemia (FHBL) (5). We have identified one IC with a compound heterozygosity in *LCAT* causing Fish Eye Disease and one IC with a variant in *ABCA1* in homozygosity causing Tangier disease. We found variants causing homozygous FHBL in 2 IC, one of whom has an undescribed pathogenic variant in homozygosity in *APOB* (c.12087+1G>A) and the other is a possible compound heterozygous for *APOB* variants c.2604+1G>A and c.4651C>T/p.(Gln1551*). In two patients only a variant in heterozygosity (c.3365delG/p.(Gly1122Vfs*62) and c.11095A>T/p.(Arg3699*)). In the remaining patient, no variants were identified. NGS proved to be a fundamental key for genetic testing of rare lipid disorders, allowing us to find the genetic cause of disease in 6/7 patients with low HDL-c and LDL-c. Patients with these rare conditions should be identified as early as possible in order to minimize or prevent clinical manifestations. The unsolved case is still under investigation.

## Introduction

Dyslipidemia is a commonly encountered clinical condition defined as an abnormal concentration of lipids in blood and ranging from raised to low plasma concentrations of total cholesterol (TC), LDL cholesterol (LDL-c), HDL cholesterol (HDL-c) or triglycerides (TGs) (Ramasamy 2016; Hegele et al., 2020). Primary dyslipidemias can be classified as hyperlipidemias or hypolipidemias, depending on whether there is an increase or decrease in plasma lipid levels. The type depends on the metabolic pathway which is affected. It is particularly important to investigate the molecular base of primary dyslipidemias since the etiology determines management and treatment for each subject affected (Hegele et al., 2020).

Primary hypobetalipoproteinemia is an inherited trait characterized by extremely low (or absent plasma) LDL-c and apolipoprotein B (apoB) concentrations. This group of diseases include Familial Hypobetalipoproteinemia (FHBL), Abetalipoproteinemia (ABL), Chylomicron Retention Disease and PCSK9 deficiency (Hegele 2013). FHBL is a rare autosomal codominant disorder characterized by low concentrations of apoB and apoB containing lipoproteins (lower than the fifth percentile for age and sex) (Moutzouri et al., 2011; Hegele et al., 2020). FHBL is caused by nonsense or frameshift variants in apolipoprotein B gene (*APOB)* leading to the formation of a truncated apoB protein of different sizes, resulting in a loss of capacity to form lipoproteins in the liver and intestine and consequently to export lipids from these organs (Ramasamy 2016). Missense variants in *APOB* gene that do not originate truncated proteins can also cause FHBL (Ramasamy 2016), but these are rare causes. Frequently, homozygous FHBL individuals have clinical manifestations such as malabsorption, deficiency of fat-soluble vitamins and acanthocytosis, atypical retina pigmentosa, neuromuscular abnormalities and hepatic steatosis. Although heterozygous FHBL also exhibit some of these manifestations, these individuals usually present a less severe phenotype (Huang et al., 1991). ABL is a rare autosomal recessive disease characterized by markedly low levels of LDL-c, triglycerides and apoB, caused by loss-of-function (LOF) alterations in the Microsomal Triglyceride Transfer Protein (*MTTP)* gene that result in truncated forms of the coded protein (Wetterau et al., 1992; Hooper et al., 2005). MTP is indispensable for the production of apoB containing lipoproteins by the liver and intestine. MTP forms a heterodimer with protein disulfide isomerase that supports the lipid transfer function. This step is important for correct folding of apoB. Due to absence of functional MTP, apoB is not correctly processed, and so the hepatic secretion of triglyceride-rich lipoproteins and fat-soluble vitamins that are transported into lipoprotein particles is impaired (Lee and Hegele 2014).

ABL patients exhibit a phenotype similar to FHBL patients being almost impossible to distinguished clinically. However, since ABL is an autosomal recessive disease, heterozygous subjects frequently have normal lipid profiles, while heterozygous FHBL patients present a characteristic phenotype with lower levels of LDL-c than the general population, consistent with a codominant inheritance mode (Lee and Hegele 2014).

Individuals with ABL or homozygous FHBL may have hepatic complications, which are however more common in individuals with FHBL. Heterozygous FHBL, can also present hepatic steatosis (Lee and Hegele 2014). The exact mechanism on the liver lipid accumulation is not clear, one idea being that the impaired lipid efflux from the liver caused by failure to assemble VLDL particles, results in chronic lipid retention (Lee and Hegele 2014).

Chylomicron retention disease, or Anderson’s disease, is a rare autosomal recessive disorder caused by biallelic loss-of-function alterations in the *SAR1B* gene that leads to failure of chylomicron secretion from enterocytes (Hooper et al., 2005; Levy et al., 2019). Often, failure to thrive is observed in childhood, along with severe malabsorption, steatorrhea, and fat-soluble vitamin deficiency (Levy et al., 2019). Homozygous patients have absence of apo B-48 and chylomicrons, and heterozygous subjects presents normal lipid profiles (Hegele et al., 2020). This *SAR1B* gene codes for a small GTPase that regulates the formation and assembly of ER-derived COPII vesicles during protein export from the endoplasmatic reticulum to the Golgi. SAR1B, as the GDP-to-GTP exchanger, is a critical element in the final step of assembling this vesicular transport complex. Consequently, alterations in this gene affect pre-chylomicron trafficking from the ER to the Golgi apparatus, leading to the absence of chylomicrons and a marked accumulation of lipids in enterocytes (Georges et al., 2011; Levy et al., 2019).

Loss-of-function variants in *PCSK9* have also been described and associated with hypocholesterolemia. The large majority are missense and nonsense alterations that prevent PCSK9-mediated LDLR degradation, and thus increase LDL uptake by the liver, resulting in a 40% reduction in plasma LDL-c levels (Lopez 2008). LOF *PCSK9* variants are not associated, until the moment, with specific clinical symptoms, and on contrary, have been associated to a lower risk of cardiovascular disease (Cohen et al., 2006).

Hypoalphalipoproteinemia is also an inherited dyslipidemia characterized by extremely low HDL-c values (<fifth percentile or <35 mg/dl). Hypoalphalipoproteinemia includes the following diseases: Tangier disease, Apolipoprotein A-I (APOA1) deficiency, and Lecithin cholesterol acyltransferase (LCAT) deficiency (including Familial LCAT deficiency (FLD) and Fish eye disease (FED)) (Hegele 2020 review). Tangier disease is inherited as an autosomal recessive trait and is caused by pathogenic variants in the *ABCA1*, which mediates the secretion of cholesterol excess from cells into the HDL metabolic pathway (Burnett et al., 2019). This results in a severe HDL deficiency and accumulation of cholesteryl esters throughout the body (Burnett et al., 2019). The major clinical signs of Tangier disease include hyperplastic yellow-orange tonsils, hepatosplenomegaly and peripheral neuropathy, which may be either relapsing-remitting or chronic progressive in nature. Rarer complications may include corneal opacities that typically do not affect vision, premature atherosclerotic coronary artery disease, occurring in the sixth and seventh decades of life (not usually before age 40 years), and mild hematologic manifestations, such as mild thrombocytopenia, reticulocytosis, stomatocytosis, or hemolytic anemia. All these clinical signs combine differently in each patient (Alshaikhli and Vaqar 2012).

APOA1 deficiency, a codominant disorder, is characterized by almost absence of HDL-c and apolipoprotein A-I (APOA-I), as well as premature coronary heart disease (Schaefer et al., 2016). To date, about 20 homozygous or compound heterozygous patients have been described worldwide with APOA1 deficiency (Hegele et al., 2020). Homozygous patients with missense variants present a very low plasma concentration of a structurally abnormal APOA-I, and can have corneal clouding, similar to Fish Eye disease (Santos et al., 2008; Schaefer et al., 2016). The homozygous patients with null alleles are clinically characterized by xanthomas, either limited to the eyelids or covering the body (Santos et al., 2008; Schaefer et al., 2016). Heterozygous patients are usually asymptomatic despite low values of HDL-c (Hegele et al., 2020).

Biallelic variants in the *LCAT* gene decrease LCAT secretion or function resulting in LCAT deficiency. LCAT is an enzyme that catalyzes the esterification of free cholesterol in HDL, thus deficiency results in accumulation of free cholesterol, but in a variability of tissues, including cornea and kidney. LCAT deficiency manifests two distinct phenotypes, FLD and FED. In FLD, alpha and beta LCAT activity is absent, leading to extremely low plasma HDL-C (below the fifth percentile). In FED, only the alpha LCAT activity is lost, the beta activity is preserved, permitting cholesterol esterification on VLDL and LDL but not on HDL (Ingle et al., 2016; Santamarina-Fojo et al., 1998). The difference between FED and FLD phenotypes seems to be on whether the variants prejudice the catalytic triad, impair the availability to the catalytic residues or affect the folding of the enzyme.

Clinical presentation of LCAT deficiency include corneal opacities, haemolytic anaemia, proteinuria, progressive chronic kidney disease (CKD), high plasma triglyceride and low HDL-cholesterol concentrations. FLD patients develop premature and progressive CKD leading to end-stage renal disease, the main cause of morbidity and mortality in these patients (Pavanello et al., 2020). Curiously, CKD is not a feature of FED patients.

Despite the profound HDL-C deficiency, LCAT deficiency does not appear to increase the risk for CVD (Zipes 2018).

The aim of this work is to present the 7 cases with low LDL and HDL cholesterol values referred to our laboratory, highlighting the most relevant clinical and molecular data.

## Material and methods

The Rare Familial Dyslipidaemia Study is a research project coordinated by the National Institute of Health (INSA) supported mainly by external funds and free of charge for all patients and health institutions. INSA Ethical Committee and INSA Data Protection Officer approved the study protocol and database. Written informed consent was obtained from all participants before their inclusion in the study.

### Biochemical characterisation of lipids and lipoproteins

Fasting blood samples were collected at the time of referral to the study. Total cholesterol, direct LDL-c, HDL-c, triglycerides TG, apoA1, apoB and lipoprotein(a) (Lp(a)) were determined for all individuals in a Cobas Integra 400 plus (Roche) by enzymatic colorimetric and immunoturbidimetric methods.

### Molecular analysis

Genomic DNA was isolated from peripheral blood EDTA samples, using an adaptation of the protocol described in D.K.Lahiri et al. (1991) (Lahiri and Nurnberger 1991).

Samples for Next-Generation Sequencing (NGS) were prepared according to SureSelect QXT Target Enrichment kit (Agilent Technologies, United States). Targeted sequencing capture probes were custom designed using the specific online tool (SureDesign) provided by Agilent*.* Only genes associated to rare forms of low HDL or LDL values were analyzed for this work, namely: *ABCA1*, *APOA1*, *LCAT, SCARB1, APOB, PCSK9, MTTP, SAR1B, and ANGPTL3*. The references used for analysis were: NM_ NM_005502, NM_000039, NM_000229, NM_005505, NM_000384, NM_174936, NM_000253, NM_001033503 and NM_014495 respectively.

The manufacturer’s protocols were followed during library preparation (Agilent Technologies, United States). In resume, for each sample, 25 ng of high-quality genomic DNA was fragmented and adaptors were added in a single enzymatic step with QXT reagents. The adaptor-tagged DNA library was purified and amplified. The libraries were recovered using streptavidin magnetic beads, and a post-capture PCR amplification was carried out. Post-enrichment pooling allowed sequencing of a high variety of sample amounts as well as different sample target sizes. Samples were pooled prior to sequencing to a final concentration of 4 nM and run in a NextSeq platform (Illumina, United States) available at INSA using a NextSeq 550 System generating 130M reads per run (75 base reads). The FASTQ files were analyzed using SureCall Software (Agilent Technologies, United States). Output VCF files were analyzed using wANNOVAR Software for SNVs identification (Yang and Wang 2015). Variants with a minor allele frequency (MAF) below 5% in gnomAD (Karczewski et al., 2019) in one of the genes studied in this work, were confirmed by Sanger sequencing.

Complementary DNA numbering was considered according to the Human Genome Variation Society (HGVS) nomenclature (Dunnen and Antonarakis 2000) with nucleotide c.1 being A of the ATG initiation codon p.1.

## Results

This study includes seven index cases, 2 with low HDL-c values and 5 with low LDL-c values. The clinical and biochemical characterization of the individuals in this study are present in Table 1.

**TABLE 1:** Clinical, biochemical and molecular characterization of the index cases in this study.

ID	Age	Gender	TC	LDL-C	HDL-C	TGs	Lp(a)	ApoA1	ApoB	Clinical diagnosis	Molecular diagnosis
1	42	F	73	46	3	158	8.3	10	91	Tangier Disease	*ABCA1* gene: c.[3460A>T];[3460A>T], p.[(Lys1154*);(Lys1154*)]
2	47	M	108	31	12	98	8.3	41	61	Fish eye Disease	*LCAT* gene: c.[619G>A];[682G>A]. p.[(Gly207Ser)];[(Asp228Asn)]
3	14	F	24	<10	22	5	9	49	10	ABL/FHBL	−
4	28	F	69	18	52	13	*	115	15	ABL/FHBL	*APOB* gene: c.3365delG:p.(Gly1122Valfs*62)
5	55	F	75^#^	27^#^	46^#^	16^#^	*	*	*	ABL/FHBL	*APOB* gene: c.11095A>T, p.(Arg3699*)
6	16	F	20	<4	29	9	8.3	82.5	<20	ABL/FHBL	*APOB* gene: c.[12,087 + 1G>A];[12,087 + 1G>A], p.(?); p.(?)
7	53	M	62	9	49	15	38	110	<20	ABL/FHBL	*APOB* gene: c.[2604 + 1G>A];[4651C>T], p.[(?)];[(Gln1551*)]

Age (years); TC–total cholesterol (mg/dl); LDL-C–low density lipoprotein cholesterol (mg/dl); HDL-C, high density lipoprotein cholesterol (mg/dl); TGs, triglycerides (mg/dl); Lp(a)—lipoprotein a) (mg/dl); ApoA1—apolipoprotein A1 (mg/dl); ApoB–apolipoprotein B (mg/dl); ABL, abetalipoproteinemia; FHBL, familial hypobetalipoproteinemia; * Not determined; # on treatment.

Four genes associated to hypoalphalipoproteinemia phenotype were analyzed (*ABCA1*, *APOA1*, *LCAT* and *SCARB1*). A total of three variants were detected, one in *ABCA1* gene (c.3460A>T, p.(Lys1154*) and two in *LCAT gene* [c.619G>A, p.(Gly207Ser) and c.682G>A, p.(Asp228Asn)] (Table 1)*.* No rare variants were identified in *APOA1* or *SCARB1* genes.

For the cases with hypocholesterolemia phenotype, we studied five genes (*APOB*, *PCSK9*, *MTTP, SAR1B,* and *ANGPTL3*). Rare variants were only detected in the *APOB* gene in four index cases (Table 1). No rare variants were identified in *PCSK9*, *MTTP*, *SAR1B*, or *ANGPTL3* genes.

### Clinical cases

#### Index case 1

A 48 years-old woman was referred to the genetic department after several myocardial infarctions (MI).

She is the first daughter of a consanguineous couple, and she has a healthy younger brother and two healthy sons. She had tonsillectomy at 4 years of age. She also has fatty liver disease and laparoscopic cholecystectomy was performed for chronic calculous cholecystitis.

She had the first MI at 42 years. At that time, the laboratory tests show TC 73 mg/dl, ApoA1 10 mg/dl and HDL-c values lower than 5 mg/dl. After cardiac exams, an atrophic right coronary artery was diagnosed, with atherosclerotic plaque burden superior than expected for age and gender.

At the age of 48 years, she had another MI and underwent angioplasty of the anterior descending coronary. Five months later, she had a third MI and a stent was implanted overlapping with the previous one.

Despite having a normal ophthalmological and neurological exam, severe deficiency of HDL and premature atherosclerotic coronary artery disease suggested Tangier disease.

**FIGURE 1:**
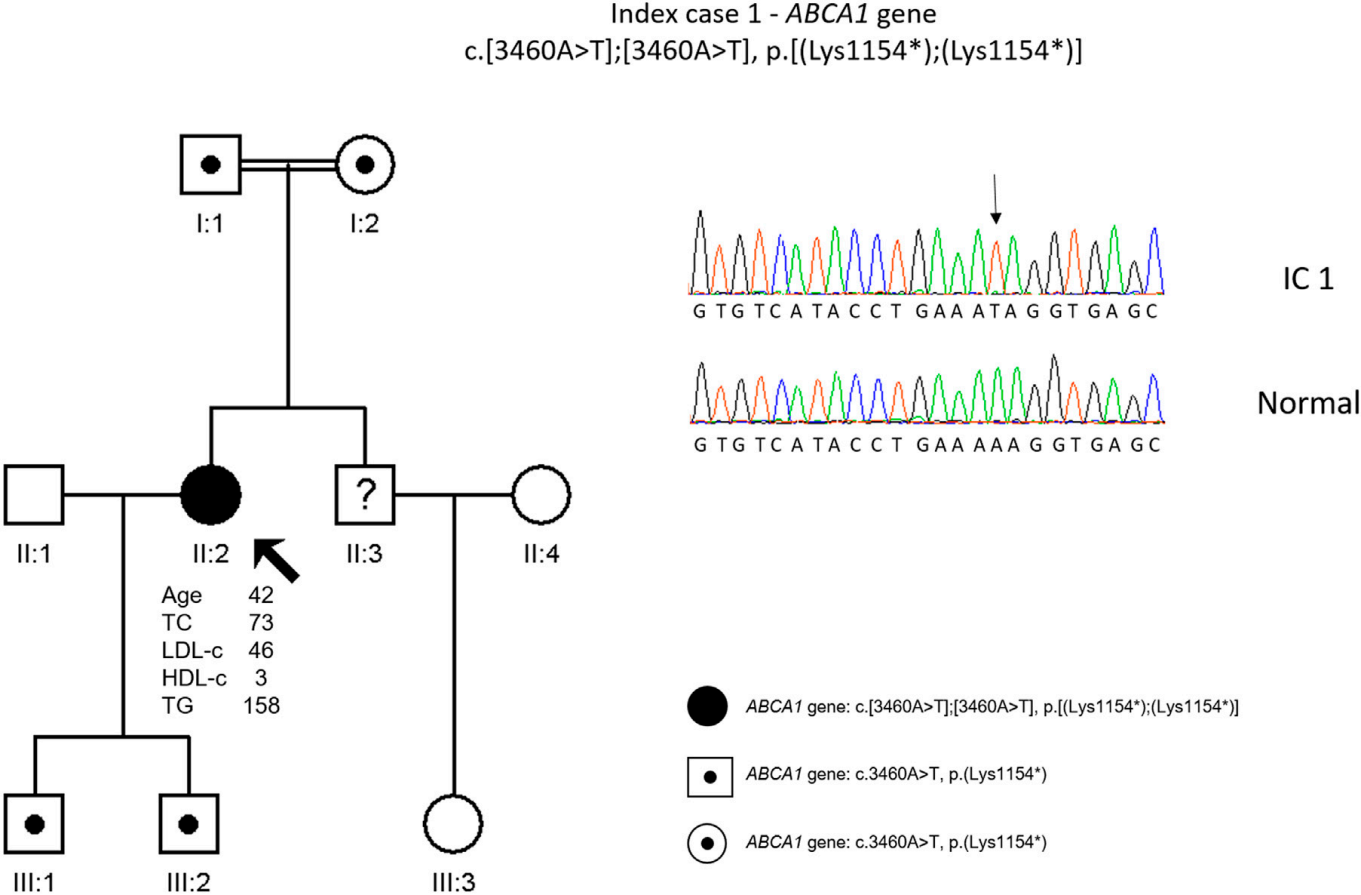
Arrow indicates index case 1 (IC1). Age (in years) and lipid profile in mg/dL are under each symbol. Point symbols represent heterozygous individuals for the variant in *ABCA1* gene, c.3460A>T, p.(Lys1154*). Black filled symbol represents homozygosity for this variant (c.3460A>T, p.(Lys1154*)). Partial sequence of *ABCA1* gene of IC1 and a normal sequence.

The genetic study revealed a homozygous variant in *ABCA1* gene, c. 3460A>T, p. (Lys1154*) (Figure 1). This variant has not been previously described but is classified as pathogenic according to the American College of Medical Genetics (ACMG) guidelines (Richards et al., 2015). No relatives were available for cascade screening, but the sons are obligatory heterozygous for the mother’s variant.

#### Index case 2

A 47-years-old male, referred from primary health center to genetic department for ocular findings combined with analytical changes, with suspected Fish-eye-disease since the age of 45. From personal medical history, there is a report of a road accident in 2008 with subsequent abdominal trauma and non-functioning right kidney.

In addition to follow-up at nephrology department, he was observed in ophthalmology, and ring opacification of the corneal periphery was revealed. Analytically, he evolved with low HDL values (6–16 mg/dl) combined with hypertriglyceridemia (158–263 mg/dl), maintained despite the fibrates therapy and diet restrictions.

**FIGURE 2:**
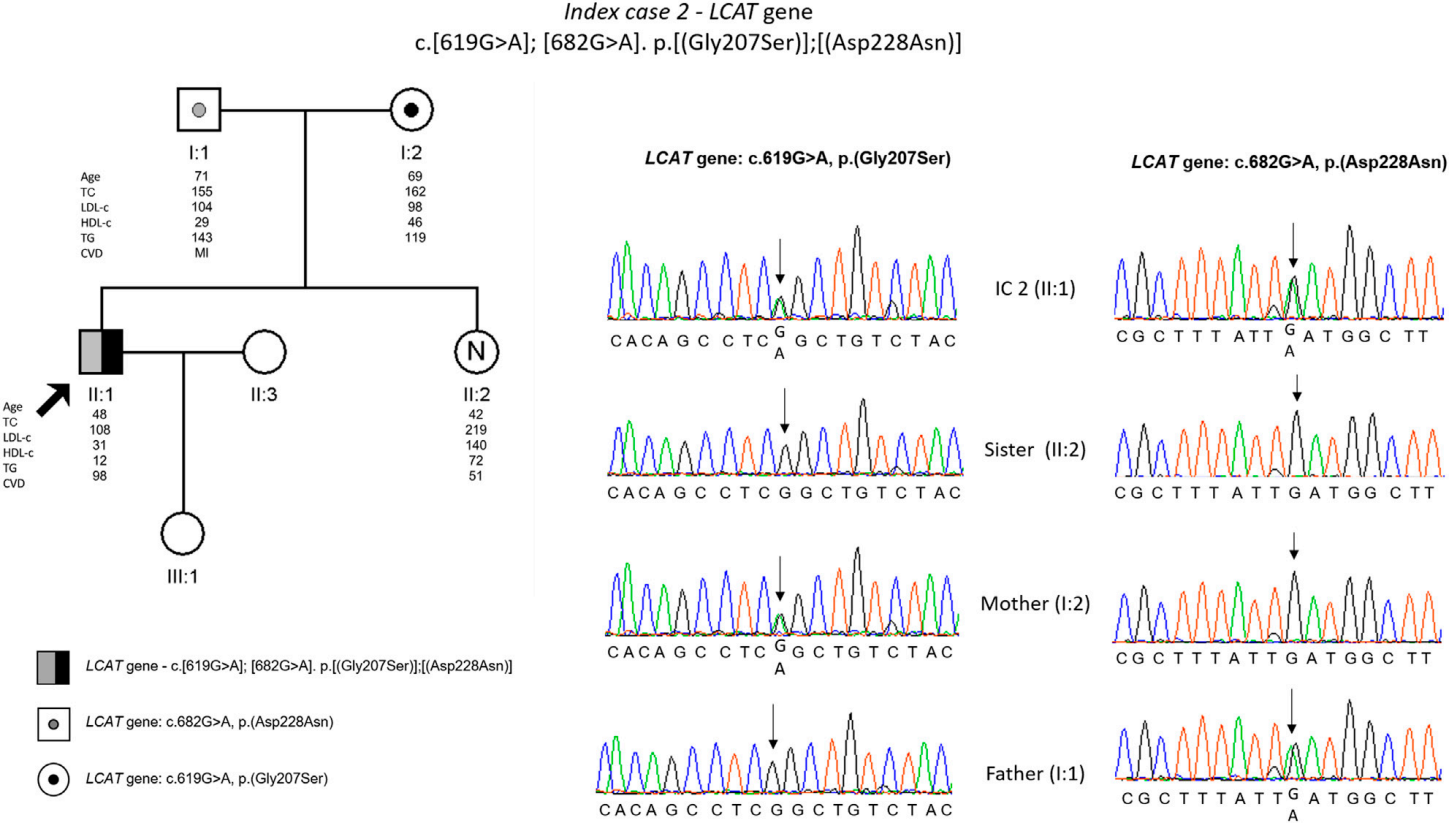
Arrow indicates index case 2 (IC2). Age (in years) and lipid profile in mg/dL are under each symbol. Black point symbol represent heterozygous individual for the variant in *LCAT* gene, c.619G>A, p.(Gly207ser); grey point symbol represent heterozygous individual for the variant in *LCAT* gene, c.682G>A, p.(Asp228Asn); the compound heterozygote is represented with half-filled in black and half-filled in grey; Partial sequence *LCAT* gene of IC2 and family members.

The genetic testing found two missense variants in *LCAT* gene: c.619G>A, p. (Gly207Ser) and c.682G>A, p. (Asp228Asn). These variants are not described previously and are classified as variants of uncertain significance (VUS) according to the ACMG guidelines. The co-segregation study was carried out on the parents, and it was observed that the index patient inherited a different variant from each parent; the sister did not carry any of the mutated alleles (Figure 2).

#### Index case 3

A 14 years-old female of Indian ancestry was referred to a pediatric consultation for poor weight and height progression. She also presented mild hepatomegaly and hyperechogenic liver suggesting liver steatosis. There was no history of chronic diarrhoea. Laboratory tests were performed showing normochromic anaemia, mild increase of transaminases and the following lipid profile: TC 24 mg/dl, LDL-c <10 mg/dl, HDL-c 22 mg/dl, TGs 5 mg/dl and apoprotein B 10 mg/dl**.**

Further investigations centered mainly on the diagnosis of a possible abetalipoproteinemia showed acanthocythosis (red blood cells with thorny spikes) on peripheral blood film, mild iron deficiency, low Vitamin D_25_, extremely low Vitamin E and A levels. Although ophthalmological examination of fundus was initially normal, later on she developed pigmentary retinopathy, in electroretinography she presented severe and progressive macular and retinal dysfunction in internal layer. Duodenal and jejunal endoscopy showed white duodenal mucosa and the Intestinal biopsy vacuolization of enterocytes (fat-filled). Follow up showed difficulty in night vision, episodes of diarrhea (initially denied) with steatorrhea and renal lithiasis. Despite very low levels of fat-soluble vitamins she presented no ataxia, hyporeflexia or muscle weakness.

Treatment included low lipid diet, high doses of vitamin A and E, as well as vitamin D and K, calcium and iron according to blood tests, showing some improvement in growth.

**FIGURE 3:**
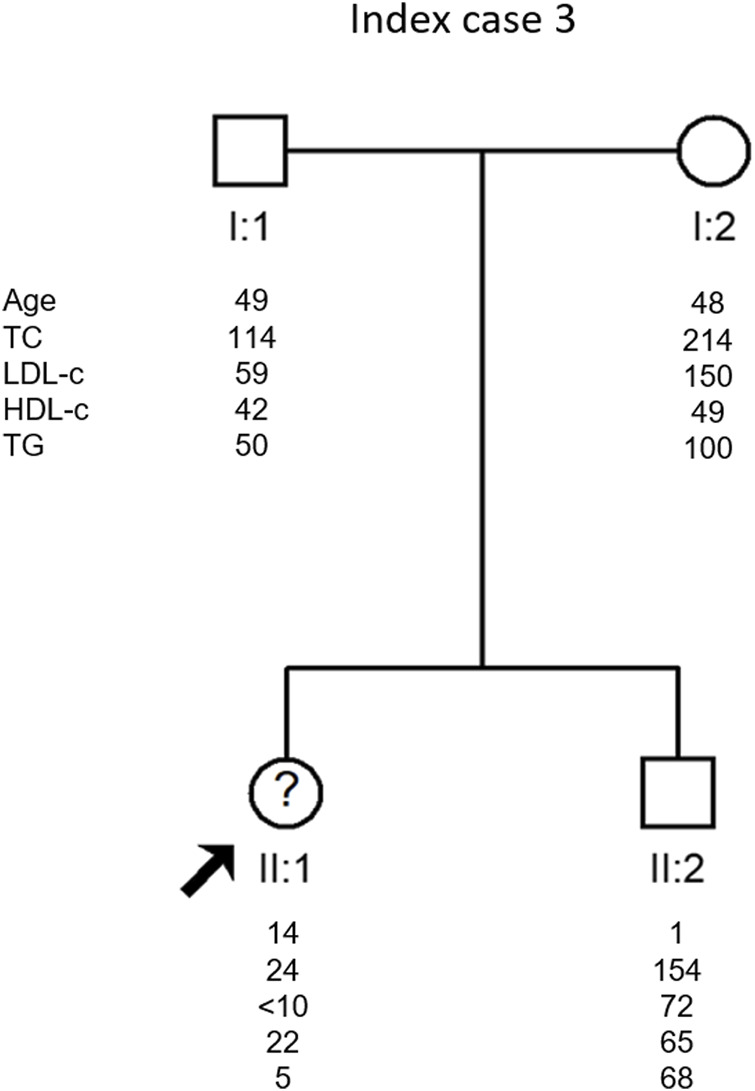
Arrow indicates index case 3 (IC3). Age (in years) and lipid profile in mg/dL are under each symbol.

Her parents have very different lipid profiles. While the father presents a typical lipid profile of heterozygous FHBL (TC 114 mg/dl, LDL-c 59 mg/dl and TGs 50 mg/dl), the mother presents a moderately high lipid profile (TC 214 mg/dl, LDL-c 150 mg/dl and TGs 100 mg/dl), but she is obese. Her brother has normal to low lipid values (TC 154 mg/dl, LDL-c 72 mg/dl and TGs 68 mg/dl) (Figure 3).

The genetic study of *APOB*, *PCSK9*, *MTTP, SAR1B,* and *ANGPTL3* genes has not detected any variant causing disease in these genes.

#### Index case 4

A 27 years-old female, was referred by an endocrinologist with hypocholesterolemia since childhood. The lipid profile was, TC 71 mg/dl, LDL-c 14 mg/dl, HDL-c 55 mg/dl, TGs 11 mg/dl and APOB <26 mg/dl. The father, paternal aunts and one of the brothers were also reported to have low total and LDL cholesterol values.

**FIGURE 4:**
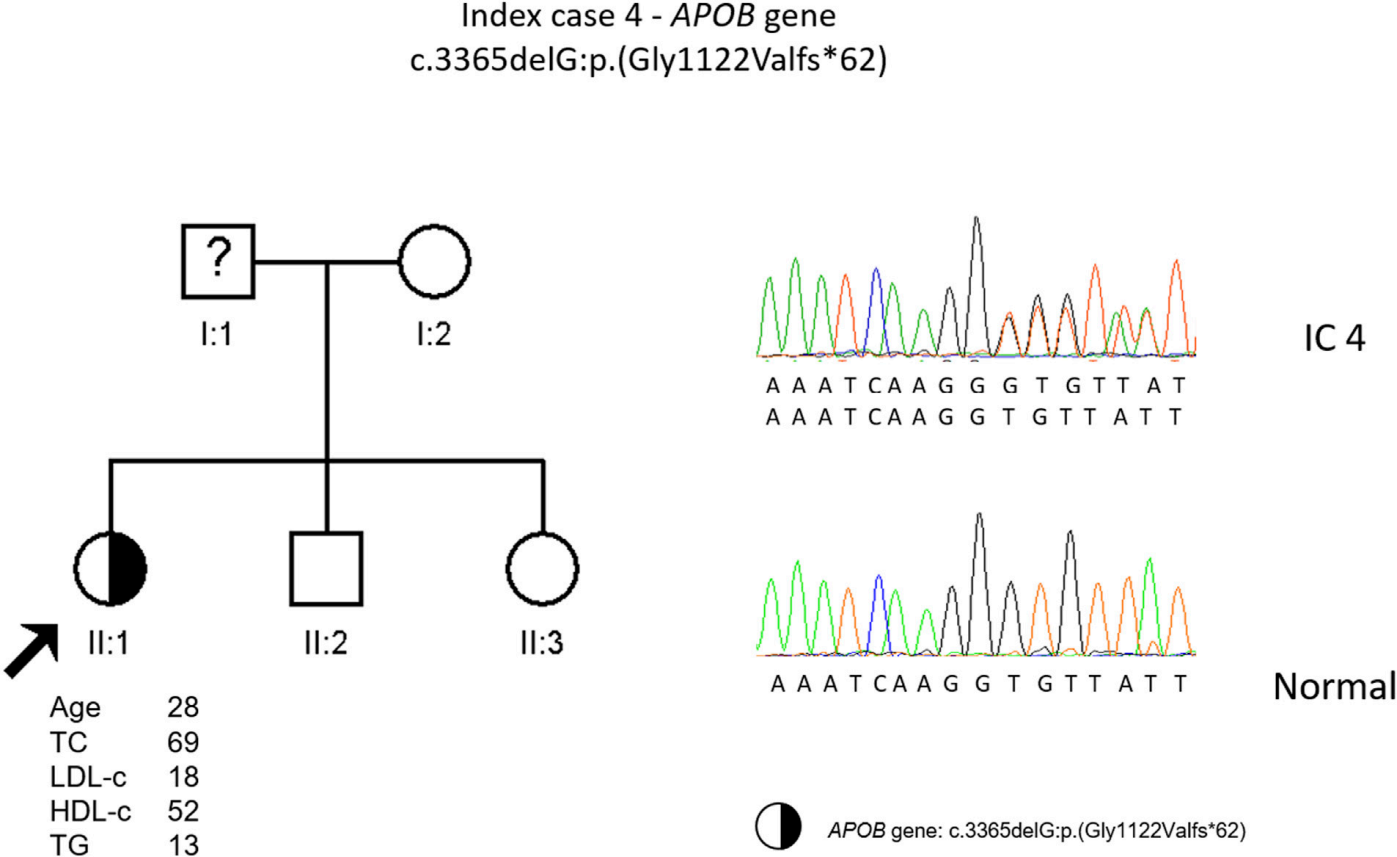
Arrow indicates index case 4 (IC4). Age (in years) and lipid profile in mg/dL are under each symbol. Half-filled symbol represents heterozygous individual for the variant in *APOB* gene, c.3365delG, p.(Gly1122Valfs*62); Partial sequence *APOB* gene of IC4 and a normal sequence.

The genetic study detected a heterozygous frameshift variant (c.3365delG, p. (Gly1122Valfs*62) in the *APOB* gene. This variant is classified as pathogenic according to the ACMG guidelines and was previously described in an individual with FHBL (Marmontel et al., 2018). It was not possible to carry out the co-segregation study in this family (Figure 4).

#### Index case 5

A 52 years-old woman with arterial hypertension and intermittent left bundle branch block in medical history, suddenly collapsed when she was working in housekeeping activities. In her family, her mother died suddenly at 48 years old, her maternal uncle also died at around 40 years and the maternal grandfather suffered sudden cardiac death at 70 years. Physical examination at the local showed no abnormalities. ECG showed a normal sinus rhythm with left bundle branch block. She was admitted to the emergency room with cranioencephalic traumatism from the fall.

An echocardiogram was performed and revealed left ventricular end-diastolic cavity at upper limits of normal and reduced left ventricular ejection fraction (LVEF) at 30–35%, with overall wall hypokinesia. She was discharged with a favourable evolution, and a cardioverter defibrillator (ICD) was implanted.

Lipid values are not available before the sudden death event. After the cardiac event she was given a statin and her LDL values reached 27 mg/dl.

**FIGURE 5:**
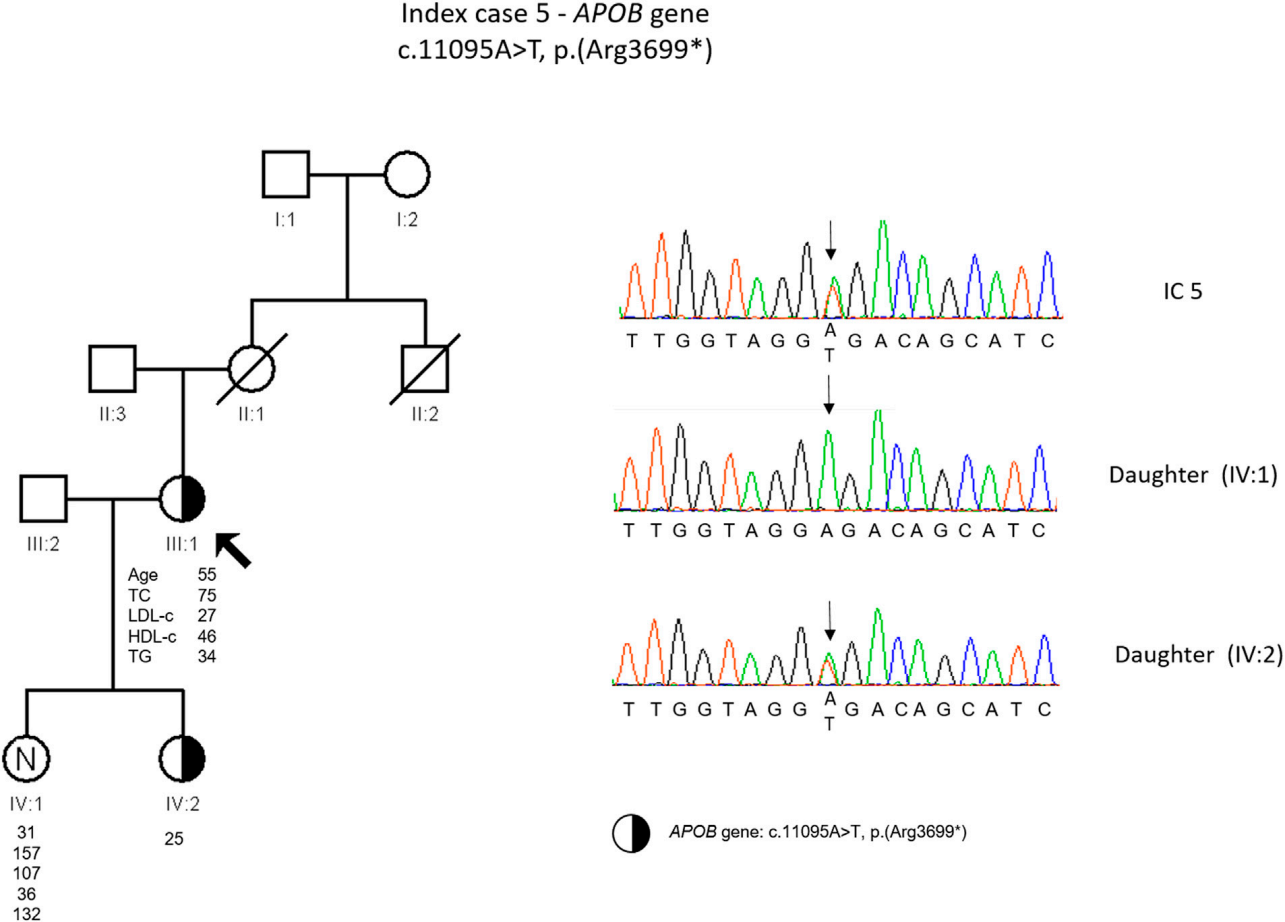
Arrow indicates index case 5 (IC5). Age (in years) and lipid profile in mg/dL are under each symbol. Half-filled symbol represent heterozygous individual for the variant in *APOB* gene, c.11095A>T, p.(Arg3699*); Partial sequence *APOB* gene of IC5 and family members

The etiological investigation with a Sudden Death Gene Panel requested by the medical geneticist to an external laboratory showed a likely pathogenic variant in *RYR2* gene (c.14579C>T). Additionally, as an incidental finding a heterozygous variant was found in the *APOB* gene, c.11095A>T, p. (Arg3699*). The presence of this variant was confirmed in our laboratory. This variant is classified as pathogenic according to the ACMG guidelines and was previously described in a heterozygous FHBL patient (Martín-Morales et al., 2013). The cascade screening performed on the index daughters revealed that one of them was carrying the *APOB* gene variant (Figure 5).

#### Index case 6

A 16-years-old female with a history of a functional specific learning disorder and family history of remote consanguinity, with no known heredofamilial diseases was admitted to Pediatric Consultation due to nephrolithiasis. On physical examination, thinness was noted (BMI 18.02 kg/m2 (P15)). “Routine” investigation disclosed TC 30 mg/dl, LDL-c 1.7 mg/dl, HDL-c 25.3 mg/dl, TGs 14.9 mg/dl (<P5 for sex and age) and mild increase of transaminases. The hypothesis of hypobetalipoproteinemia was supported by apolipoprotein B100 < 12 mg/dl. Neurological and ophthalmological exams were normal. Liver ultrasound was also normal (no steatosis).

The dosage of vitamins A, D and E showed deficits in vitamin D (22 ng/ml; r.v.>29) and E 4.25 mg/L (r.v. 5–20). The coagulation study showed an INR of 1.28 (r.v. ≤1.2) with factor VII 41% (r.v. 50–150). At this stage she started vitamin E, D and K supplementation.

**FIGURE 6:**
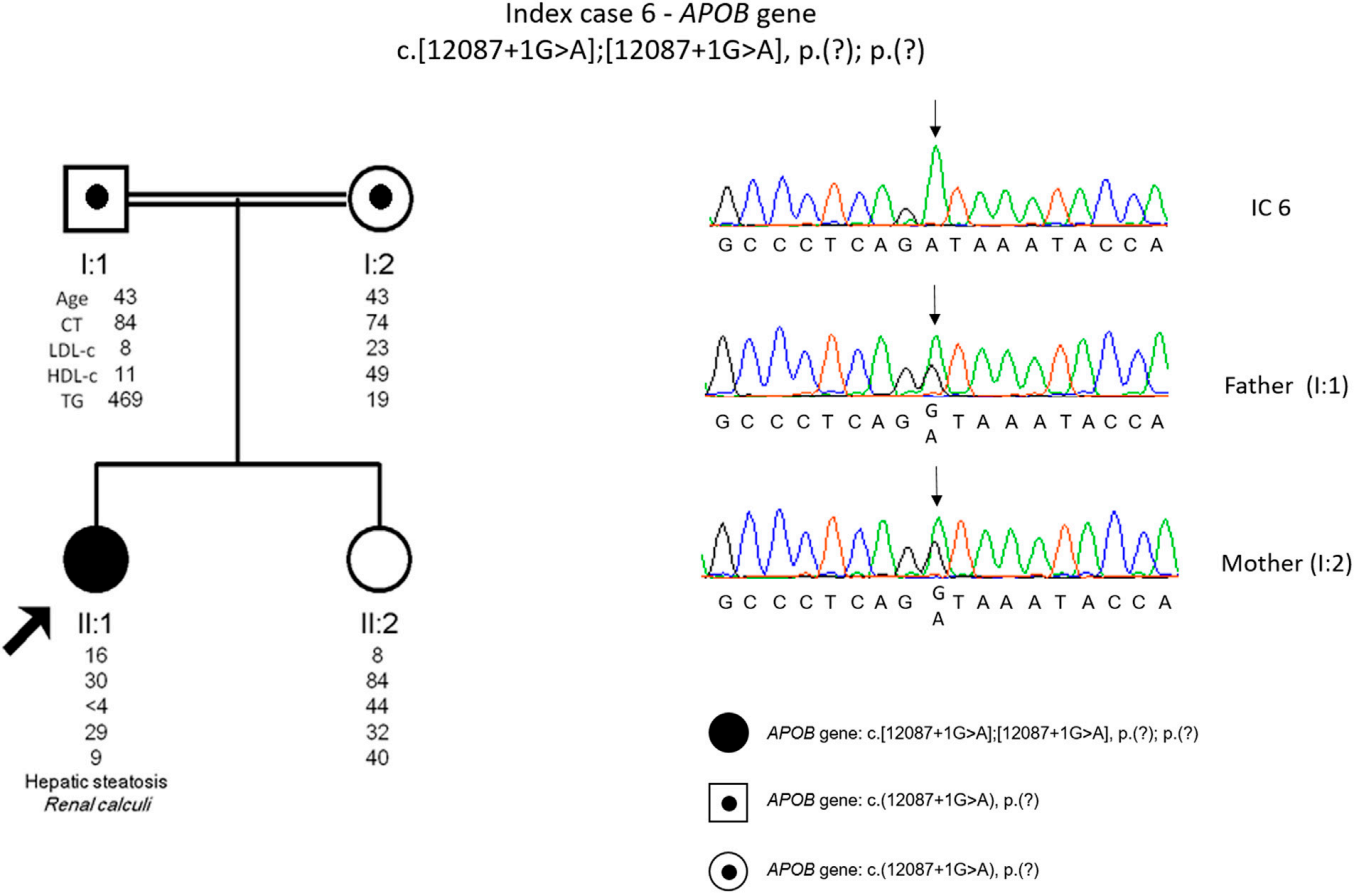
Arrow indicates index case 6 (IC6). Age (in years) and lipid profile in mg/dL are under each symbol. Black point symbol represents heterozygous individual for the variant in *APOB* gene, c.12087+1G>A, p.(?); Black symbol represent homozygous individual for the variant in *APOB* gene, c.12087+1G>A, p.(?); Partial sequence *APOB* gene of IC6 and family members.

The genetic study detected a homozygous variant [c.12087 + 1G>A, p. (?)] in the *APOB* gene. This variant is novel and is classified as pathogenic according to the ACMG guidelines. The cascade screening was carried out on the parents, and it was found that the parents are both carriers of the variant in the *APOB* gene (Figure 6).

#### Index case 7

A 53 years-old male with a previous history of follicular variant papillary carcinoma that was removed, was referred to our lab by an internal medicine physician and presented with hypocholesterolemia, hepatomegaly, steatosis hepatic and arterial hypertension. The lipid profile was TC 62 mg/dl, LDL-c 9 mg/dl, HDL-c 49 mg/dl, TGs 15 mg/dl and mild increase of transaminases. Neurological and ophthalmological exams were normal.

**FIGURE 7:**
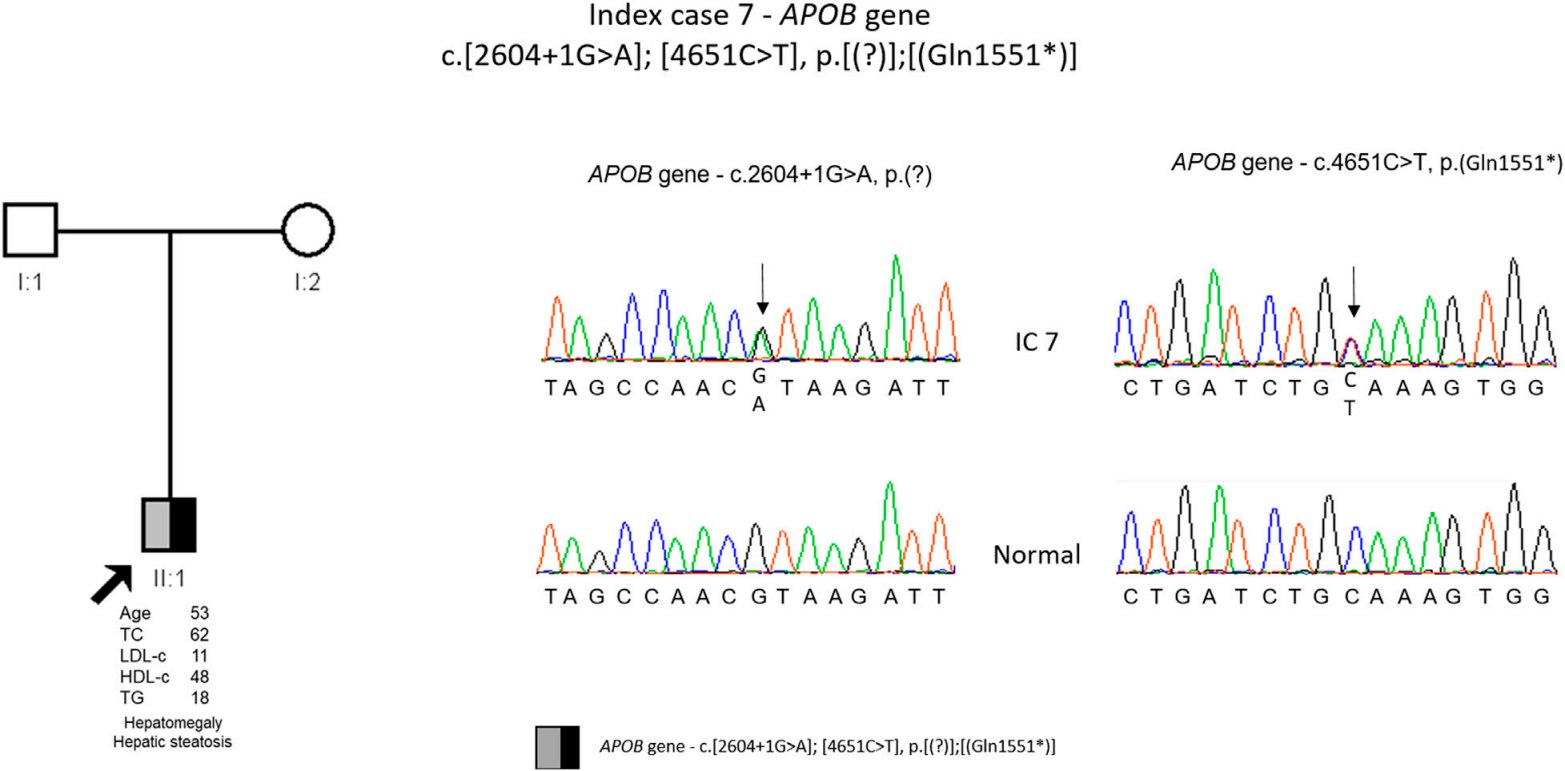
Arrow indicates index case 7 (IC7). Age (in years) and lipid profile in mg/dL are under each symbol. Black half-filled symbol represent heterozygous individual for the variant in *APOB* gene, c.2604G>A, p.(?); Grey half-filled symbol represent heterozygous individual for the variant in *APOB* gene, c.4651C>T, p.(Gln1551*); Partial sequence *APOB* gene of IC7 and normal sequence.

The genetic study detected two variants in *APOB* gene, c.2604 + 1G>A, p. (?) and c.4651C>T, p. (Gln1551*). Both variants were described previously, the splicing variant by Pelusi and others (Pelusi et al., 2019) and the nonsense variant by Lange and others (Lange et al., 2014). These variants are classified as pathogenic according to the ACMG guidelines. The cascade screening could not be performed until now due to parents old age and reduced mobility (Figure 7).

### Discussion

Serious clinical complications resulting from genetic dyslipidemias awakened the scientific community for the need to investigate these disorders.

With the advent of NGS technology, massive parallel DNA sequencing became possible, as well as the rapid and cost-effective sequencing of the entire genome hence altering the traditional laboratory approach to genetic testing and research (Sanger Sequencing) (Shendure et al., 2017). As a research tool, NGS customized panels allow a focused investigation of rare familial dyslipidemias by combining genes associated with familial dyslipidemias and genes that are candidates for an important role in lipid metabolism, allowing for a quick analysis.

In this work we present the clinical and molecular data of seven index cases (ICs), clinically diagnosed with rare dyslipidaemias: hypoalphalipoproteinemia (2 ICs) and hypocholesterolemia (5 ICs).

#### Hypoalphalipoproteinemia

Patient 1 with a nonsense variant in homozygosity in *ABCA1* gene [c.3460A>T, p. (Lys1154*)] presented an unusual phenotype having had several MI around 40 years old. Although the development of CHD is a characteristic of the disease, this patient presented it at an early age (Hegele et al., 2020). Furthermore, she has fatty liver disease and laparoscopic cholecystectomy was performed for chronic calculous cholecystitis. Although at the moment, there is no specific treatment for this disease, early identification could have prevented the development of CHD at such an early age. The therapeutic options are to increase HDL levels through lifestyle alterations such as physical exercise, a healthy weight, smoking cessation, and replacement of monounsaturated for saturated fatty acids in diet, in order to raise HDL cholesterol. In some cases, a combination of lipid-lowering therapies such as statins, niacin, and fibrates can be given either alone or in combinations to optimize LDL levels (Hegele et al., 2020; Alshaikhli and Vaqar 2012). However, there is no robust evidence of the efficacy of this therapeutic approach (Alshaikhli and Vaqar 2012). In this patient, LDL levels were already low and this approach was not chosen.

Cascade screening was not carried out but, since this patient is a true homozygous patient, her sons and parents are obligatory carriers of this variant. No health complications are predicted since they will present this variant in heterozygosity, however, for future reproductive issues the index case offspring should be offered genetic counseling since they are carriers of a pathogenic variant that in homozygosity is associated with a high cardiovascular risk.

The second patient with hypoalphalipoproteinemia presented ring opacification of the corneal periphery since childhood, characteristic of Fish Eye Disease (Hegele et al., 2020). However, the diagnosis was only made later in life. The molecular study confirmed the presence of two different variants in the *LCAT* gene, both not previously described. Parents are unrelated and so the compound heterozygosity was expected in this patient. It is described that heterozygous have HDL-cholesterol levels about half of reference values (Hegele et al., 2020), and in fact the IC parents have low HDL values, most clearly the father (29 mg/dl). As expected, the non-carrier sister has a normal HDL value (74 mg/dl).

Most patients with FED have moderately high triglyceride levels (Ramasamy 2016), since they have high levels of VLDL, TG enrichment of LDL and reduction of HDL (Schaefer et al., 2016). Our patient presented mild hypertriglyceridaemia and is receiving appropriate pharmacological treatment with fibrates.

At 35 years old he had a road accident, resulting in only one functioning kidney. As kidney disease is not associated with FED patients (Santamarina-Fojo et al., 1998), this issue will not affect his prognosis.

#### Hypocholesterolemia

We identified a rare variant in the *APOB* gene in four out of five index cases with a clinically diagnosis of hypocholesterolemia.

IC three was the only case where the cause of hypocholesterolemia has not yet been identified. All genes associated to date with hypocholesterolemia have been studied and no disease-causing variant has been identified. This is one of the most enigmatic cases in our laboratory since she presents a severe hypocholesterolemia phenotype but no variant explaining the phenotype was found. Moreover, her mother seems to have a mild dyslipidemia which is not common in relatives of patients with hypocholesterolemia. In this family, a whole exome sequencing will be performed in an attempt to find the cause of hypocholesterolemia.

Patient four is also an interesting case. After the genetic study to investigate the cause of an episode compatible with the sudden death syndrome, a frameshift variant in the *APOB* gene was found as an incidental finding. This variant, c.3365delG, p. (Gly1122Vfs*62), causes an alteration in the reading frame resulting in a premature termination codon in *APOB*, not described previously. The predicted translation product with this alteration is a truncated protein, smaller than apoB-48. Due to a family history of sudden death and the finding of an alteration in *RYR2* gene variant, this was considered the cause of the disease and a statin was prescribed. In this case, being the patient heterozygous for FHBL, lipid lowering therapeutics should not be necessary since the patient naturally has low LDL values. One of the daughters is also heterozygous for a frameshift variant in the *APOB*. We do not have information about plasma levels, but we assume they will be low. Both mother and daughter should be evaluated for fatty liver disease due to lack of apoB protein produced from one allele.

IC 5 has the diagnosis of heterozygous FHBL, since a non-sense variant in the *APOB* gene in heterozygosity resulting in a premature stop codon consequently generating a truncated protein [c.11095A>T, p. (Arg3699*)] was identified. This variant has been described previously in heterozygosis, in a Spanish patient with a clinical diagnosis of FHBL (Martín-Morales et al., 2013). However, the plasma levels in our patient are lower compared to that described by Martim-Morales and others (Martín-Morales et al., 2013). In both families, there are relatives with low and normal LDL levels and there is no report of neurological disease or fatty liver. Nevertheless, the carriers of this variant should be followed in a specialized center to prevent the long-term complications.

IC 6, a definite diagnosis of homozygous FHBL was possible to achieve since the patient presents a novel splicing variant in homozygosity that justifies the phenotype. Following the co-segregation study, it was found that the *APOB* gene variant was indeed inherited from each parent. The parents have remote consanguinity, justifying the presence of the variant in true homozygosity. To date it was not possible to perform the co-segregation study on the sister, but according to the lipid profile (LDL 44 mg/dl) she seems to be a carrier of the variant in the *APOB* (Figure 6). Although the patient has the most severe form of the disorder (homozygous form), she is in a good clinical condition without neurological symptoms, although with low levels of fat-soluble vitamins. Supplementation should prevent long-term complications, such as ataxia and peripheral neuropathy. Fatty liver, not found in the patient, should be investigated in the heterozygous parents. Considering the rarity and heterogeneity of the clinical severity of familial hypobetalipoproteinaemia, this diagnosis is a challenge in terms of therapeutic and clinical surveillance.

In IC 7, two *APOB* variants were identified, one splicing variant [c.2604 + 1G>A. p. (?)] and other non-sense [c.4651C>T, p. (Gln1551*)]. Unfortunately, the cascade screening could not be carried out in this family due to the old age of the parents who live in the islands and therefore it was not possible to confirm that the IC is a compound heterozygous. However, due to the patient phenotype there is little doubt that each variant is in a different allele. The non-sense variant creates a premature codon and consequently a truncated protein and the splicing variant, considering that is at position +1, it is in a critical location for the correct RNA splicing and most probably will disrupt RNA splicing. This will result in exon skipping or intron retention that in turn will lead to frameshift originating a truncated protein. Furthermore, both variants have been described previously in individuals with a clinical diagnosis of FHBL (Lange et al., 2014; Marmontel et al., 2018). So, both variants are considered disease causing. This patient presents hepatic steatosis, and interestingly the splicing variant was previously described in an Italian patient with non-alcoholic fatty liver disease (NAFLD) and hepatocellular carcinoma (NAFLD-HCC) (Pelusi et al., 2019). Pelusi and others observed that individuals with NAFLD-HCC and *APOB* variants had a circulating lipid profile consistent with hypobetalipoproteinemia (Pelusi et al., 2019). It was also described that variants in *APOB* also frequently occur during hepatic carcinogenesis since there is a causal role of hepatocellular lipid retention in promoting NAFLD-HCC (Ally et al., 2017). The mechanism that relates *APOB* variants with carcinogenesis is not well understood. Some of the hypotheses are induction of hepatocellular lipid accumulation, oxidative stress, and the loss of a possible tumor suppressive activity of *APOB* (G. Lee et al., 2018; Valenti and Romeo 2017).

Functional assessment of NGS detected variants in these cases is relevant to understand the effect of these specific truncations since the length of the truncated protein may have clinical significance (Richards et al., 2015). All variants found in the *APOB* gene are predicted to prevent the complete translation of apoB mRNA, resulting in the production of truncated dysfunctional apoB proteins that are most probably degraded by the cell. Without apoB, new lipoproteins are not formed in the liver and/or intestine and exported, resulting in low or absent LDL levels in plasma (Sankatsing et al., 2005; Rabacchi et al., 2019). Liver steatosis in FHBL individuals might be explained by chronic lipid retention due to decreased production of LDL apo B-100, increased catabolism of VLDL, and extremely low secretion of the truncated apo-B (Parhofer et al., 1992). The screening of liver steatosis in these patients is seldom done in the clinical set, especially in heterozygous cases, preventing early diagnosis and treatment.

In addition, cascade screening is essential to study these inherited disorders in order to access variant pathogenicity and to early identify affected relatives who might benefit from implementation of therapeutic measures. Particularly in FHBL, due to its co-dominant inheritance pattern, heterozygous relatives are at risk and should be offered genetic assessment and counselling (Hegele et al., 2020).

Studies focused on FHBL subjects demonstrated by correlation that they are relatively protected from CVD by the (life-long) reduced levels of exposure to apo B-containing lipoproteins, suggesting that apo B-containing particles may constitute a central factor in atherogenesis (Sankatsing et al., 2005). Thus, FHBL patients might constitute a unique cohort to evaluate the impact of life-long exposure to unusually low levels of apoB-containing atherogenic lipoproteins. Non-etheless, despite low cardiovascular risk of FHBL patients, other serious conditions are associated with this disorder, such as neurological complications and steatohepatitis (J. Lee and Hegele 2014).

## Conclusion

We report here the clinical and molecular characteristics of seven patients with rare lipid disorders associated with low LDL or low HDL. Two cases presented with hypoalphalipoproteinemia, and the genetic workup allowed the diagnosis of Tangier disease in one and of Fish Eye Disease in the other. These are ultrarare disorders, with 200 and 30, cases respectively reported worldwide (www.orphanet.com).

Five out of seven patients had a clinical diagnosis of hypocholesterolaemia and variants in *APOB* were identified in 4. Half have the milder form of FHBL (heterozygous form), but also need to be counselled since they are at risk for liver steatosis. In the only patient without molecular diagnosis in our cohort, extended molecular studies (eg exome) will be performed to find the cause of the severe hypocholesterolaemia.

This work shows that NGS custom panels allow for a rapid and accurate identification of patients with rare dyslipidaemia. When the cause is not found, novel genes can be investigated by exome/genome sequencing. (Santamarina-Fojo et al., 1998).

## Data Availability

The data presented in the study are deposited in the NCBI repository, accession numbers are: OQ692142 and OQ692158. The data can be accessed here: https://www.ncbi.nlm.nih.gov/nuccore/OQ692142 and https://www.ncbi.nlm.nih.gov/nuccore/OQ692158
